# Visible Light Responsive Photocatalyst Induces Progressive and Apical-Terminus Preferential Damages on *Escherichia coli* Surfaces

**DOI:** 10.1371/journal.pone.0019982

**Published:** 2011-05-12

**Authors:** Je-Wen Liou, Ming-Hui Gu, Yen-Kai Chen, Wen-Yi Chen, Yi-Cheng Chen, Yao-Hsuan Tseng, Yu-Jiun Hung, Hsin-Hou Chang

**Affiliations:** 1 Department of Biochemistry, Tzu Chi University, Hualien, Taiwan, Republic of China; 2 Institute of Medical Sciences, Tzu Chi University, Hualien, Taiwan, Republic of China; 3 Nanotechnology Research Center, National Dong-Hwa University, Hualien, Taiwan, Republic of China; 4 Department of Laboratory Medicine and Biotechnology, Tzu Chi University, Hualien, Taiwan, Republic of China; 5 Department of Chemical Engineering, National Taiwan University of Science and Technology, Taipei, Taiwan, Republic of China; 6 Department of Molecular Biology and Human Genetics, Tzu Chi University, Hualien, Taiwan, Republic of China; Indian Institute of Science, India

## Abstract

**Background:**

Recent research shows that visible-light responsive photocatalysts have potential usage in antimicrobial applications. However, the dynamic changes in the damage to photocatalyzed bacteria remain unclear.

**Methodology/Principal Findings:**

Facilitated by atomic force microscopy, this study analyzes the visible-light driven photocatalyst-mediated damage of *Escherichia coli*. Results show that antibacterial properties are associated with the appearance of hole-like structures on the bacteria surfaces. Unexpectedly, these hole-like structures were preferentially induced at the apical terminus of rod shaped *E. coli* cells. Differentiating the damages into various levels and analyzing the percentage of damage to the cells showed that photocatalysis was likely to elicit sequential damages in *E. coli* cells. The process began with changing the surface properties on bacterial cells, as indicated in surface roughness measurements using atomic force microscopy, and holes then formed at the apical terminus of the cells. The holes were then subsequently enlarged until the cells were totally transformed into a flattened shape. Parallel experiments indicated that photocatalysis-induced bacterial protein leakage is associated with the progression of hole-like damages, further suggesting pore formation. Control experiments using ultraviolet light responsive titanium-dioxide substrates also obtained similar observations, suggesting that this is a general phenomenon of *E. coli* in response to photocatalysis.

**Conclusion/Significance:**

The photocatalysis-mediated localization-preferential damage to *E. coli* cells reveals the weak points of the bacteria. This might facilitate the investigation of antibacterial mechanism of the photocatalysis.

## Introduction

Disinfectants are important to reduce the number of pathogenic microorganisms for critical instrument sterilization, water treatment, food production, and hospitals or health care facilities. Most commonly used disinfectants are chemical-based. These disinfectants, such as alcohols, iodine, and chlorine, have been used for hundreds of years. Unlike chemical based disinfectants, photocatalyst-based disinfectants are relatively novel and still under development. Photocatalytic ultraviolet (UV) light responsive titanium dioxide (TiO_2_) substrates can effectively eliminate organic compounds or work as disinfectants [1]–[2]. Upon excitation by UV light, the photon energy creates pairs of electron and hole that diffuse and become trapped on or near the TiO_2_ surface. The electrons and holes generated by these reactions have a strong reducing and oxidizing effect, and subsequently react with atmospheric water and oxygen to yield active oxygen species (ROS), such as hydroxyl radicals (.OH) and superoxide anions (O^2−^) [3]. Both holes and ROS are extremely reactive when contacting organic compounds [3]–[4]. However, since UV irradiation is hazardous to humans. UV-responsive TiO_2_ photocatalysts are unsuitable for applications in indoor environments. Recent reports demonstrate that doping TiO_2_ with impurities such as carbon, sulfur, nitrogen or silver, results in excitation wavelength shifts from UV to the visible light region [5]–[9], while the doped substrates still exhibit effective anti-microorganism activities [5]–[9]. However, the molecular mechanism and key cellular targets of the photocatalysis remain unclear. Since photocatalytic reactions generate both oxidizing and reducing activity [3]–[4], the damage they cause to target microorganisms should be very different from those caused by existing disinfectants with either oxidizing or reducing activity alone. Bacterial membrane lipids are a target of photocatalysis [1]. There is also evidence that bacterial proteins are important targets, as photocatalysis inactivates bacterial exotoxins, and thereby reduces their pathogenicity [10]. However, the real action mode and direct visualization of the photocatalytic process on bacterial surfaces remain unclear. To gather information regarding the bactericidal mechanism of photocatalysts, it is first necessary to capture the changes on bacterial surfaces during photocatalysis at sufficient resolution.

This study uses atomic force microscopy (AFM) [11]–[13] to analyze the morphological and surface changes of *E. coli* cells during photocatalyst treatments. The major advantage of AFM is that it permits high-resolution visualization of cells *in situ* without harsh chemical or physical treatments, as compared with scanning electron microscopy (SEM). This makes it a suitable tool to study the mechanisms of photocatalysis on target bacteria. The antibacterial experiments in this study compared a newly developed carbon-doped visible light responsive TiO_2_ substrate, C200 nanopowder [14]–[17], to UV-responsive UV100 TiO_2_ substrate [14]. The analytical atomic force microscopic technique was applied to investigate the initial stages of the sterilization effect of C200 on the nano-scale on *E. coli* surfaces. Time dependent photocatalysis-mediated surface changes on *E. coli* were recorded. This study also discusses the potential mechanism on the bacterial inactivation.

## Results

### Antibacterial properties of C200 photocatalysts under visible light illumination

**Figure 1:**
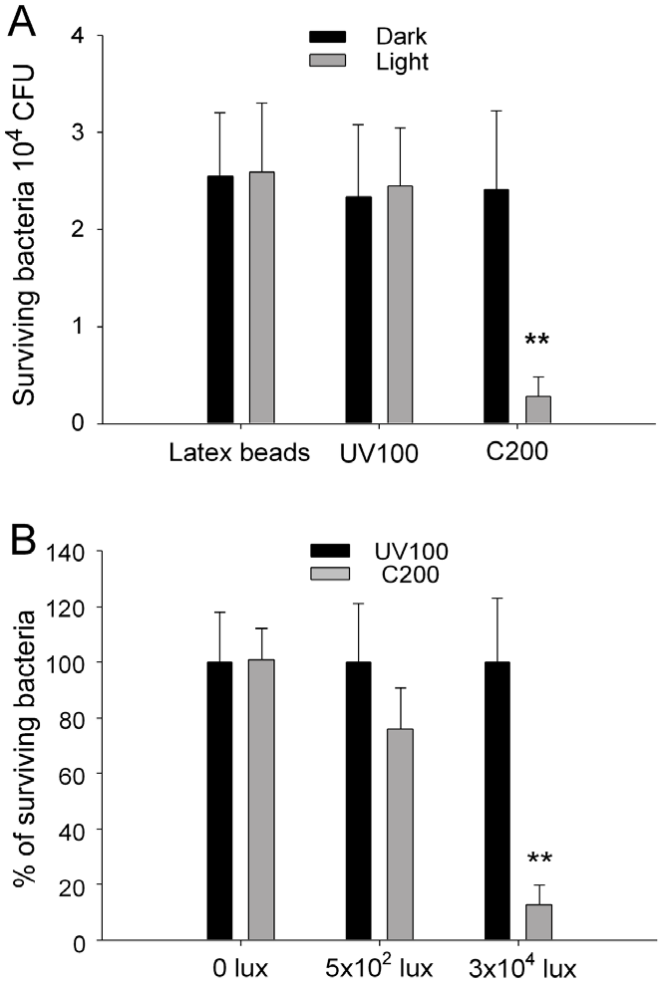
Antibacterial properties of UV light responsive photocatalyst UV100 and visible light responsive photocatalyst C200 under visible light illumination. (A) Antibacterial properties of UV100 and C200 photocatalysts against *E. coli*. Illumination was produced by a classical incandescent light bulb at a light density of 3×10^4^ lux (90 mW/cm^2^) for 5 min (“light” groups). The control latex beads and the commercialized UV-responsive photocatalyst UV100 showed no antibacterial effect under visible light illumination. Only the visible-light responsive C200 photocatalyst groups showed a significantly reduced number of bacteria after illumination (** *P*<0.01, compared to both latex beads and UV100 groups). The “dark” groups were the bacteria prepared in the same conditions without illumination (A). CFU: colony forming unites. (B) Dose dependency analysis of the bactericidal activity of the C200 substrate after visible-light illumination. Illumination was carried out with different light densities for 5 min. The number of surviving bacteria in UV100 groups in each illumination condition was normalized to 100%. ** *P*<0.01 compared to respective UV100 groups.

Antibacterial property of visible light-responsive photocatalyst C200 was reproduced and compared with a UV-responsive photocatalyst UV100 and control latex beads (Fig. 1A). In agreement with previous research [14], the C200 photocatalyst significantly inactivated *E. coli* cells under visible light illumination compared with the two control materials (C200 vs. UV100 and latex beads groups, Fig. 1A). The C200-treated bacteria showed no significantly decrease of the viable cells in the dark, indicating that C200 cannot inactivate bacteria without illumination (Fig. 1A, C200 vs. latex beads, UV100, without illumination groups). The bactericidal activity of C200 was dose dependent in response to various illumination densities of visible light, and the antibacterial effect was significant at a dose of 3×10^4^ lux, compared to UV100 3×10^4^ lux groups (90 mW/cm^2^) (Fig. 1B, ** *P*<0.01).

### Atomic force microscopy (AFM) topographic imaging of C200-photocatalyzed *E. coli*

**Figure 2:**
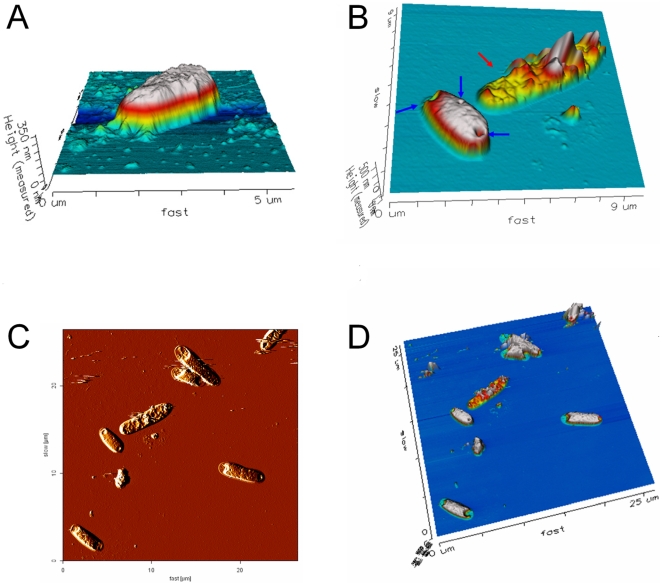
Representative AFM topographic images of photocatalyzed *E. coli* cells. The AFM images showed the cellular morphology of *E. coli* that was treated before (A) and after (B–D) C200-photocatalysis (visible light 1 minute). To imitate a three-dimensional image, this figure presents the height information as pseudo-color (A, B, D). Compared to the control cell (A), C200-mediated photocatalysis greatly damaged certain bacteria, which appeared to be flattened (red arrow in B). Nevertheless, many of the bacteria cells were only partially damaged and many of them showed signs of beginning stage of the deformations. In these cases, holes are found on the cell surfaces (blue arrows in B). This figure also provides lower magnification views of Fig. B (C, D). A two-dimensional vertical deflection image could show the best contrast and provide a more clear detail on the top of the cells (C). The height information indicated in figure D. Most of the bacterial damage appeared near the poles of the rod-shape bacteria (C, D).

This study uses AFM imaging to investigate the surface changes of photocatalyzed bacteria. Figure 2 shows representative images of *E. coli* cells in untreated (Fig. 2A) and 1 minute C200-photocatalyzed groups (Fig. 2B–D, visible light 90 mW/cm^2^). Compared with the control cells (Fig. 2A), a considerable number of the bacteria exhibited initial stages of deformation (blue arrows in Fig. 2B). Some of cells were badly damaged, exhibiting a flattened shape after photocatalysis for 1 minute (red arrow in Fig. 2B). Hole-like structures appeared on the surfaces of cells with initial damages (blue arrows in Fig. 2B). Compared with more cells in lower magnification images, these hole-like deformations seemed to be preferentially induced from the apical terminuses of the bacteria (Fig. 2C, D).

### Bacterial protein leakage indicates pore formation after photocatalysis

**Figure 3:**
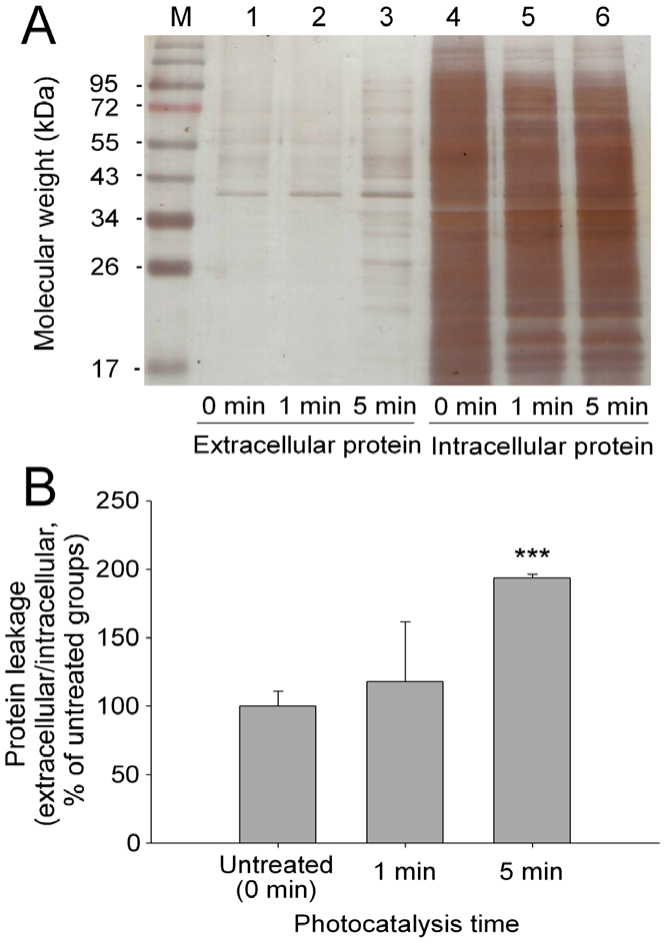
Measurement of bacterial protein leakage by SDS-PAGE. (A), The SDS-PAGE gel image indicated the extracellular bacterial proteins (leaked into the medium, lane 1–3) and intracellular bacterial proteins (lane 4–6) before (control, lane 1, 4) and after C200-photocatalysis (visible light) for 1 minute (lane 2, 5) and 5 minutes (lane 3, 6), respectively. One representative image out of three experiments is shown. Total protein in the cells after the treatments was used as an internal control for quantification (lane 4–6). The numbers on the left indicate the molecular weights of the marker proteins in units of kDa. (B) The quantitative results of SDS-PAGE. The quantification was based on intensity measurements on the whole lanes using Image J software. The average protein levels in lanes 1–3 were first divided by the average protein levels in lanes 4–6, respectively, and then the ratio (lane 1/lane 4) of untreated groups was normalized to 100%. *** *P*<0.001, compared to the untreated control group.

The hole-like images in this study might only reflect a structural change in the bacterial surfaces, and not the formation of a real pore. Cellular proteins are relatively large components compared with the small molecules and ions in the cells. Thus, the leakage of cellular protein may represent the severity of pore formation. Therefore, protein leakage from the photocatalyzed bacteria (log phase) was then analyzed and measured using SDS-PAGE. Results show that bacterial proteins were released from the cells in a time dependent manner (Fig. 3; visible light 90 mW/cm^2^; untreated vs. 5 min groups, *** *P*<0.001), suggesting that the visible-light driven photocatalysis produced hole-like damages on the bacterial surfaces.

### Atomic force microscopy analysis of the surface roughness of C200-photocatalyzed *E. coli* cells

**Figure 4:**
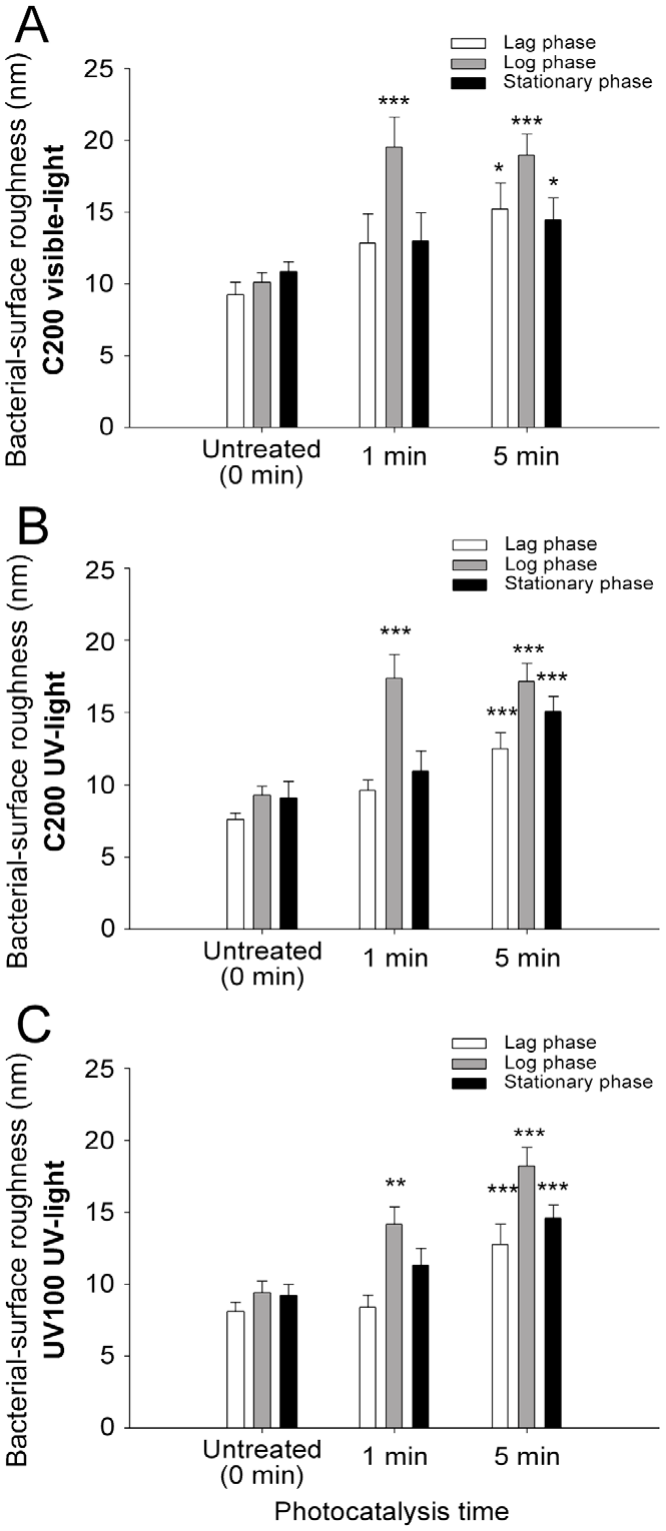
Surface roughness of *E. coli* cells before and after photocatalytic treatment with the C200 measured by AFM. The root mean square deviation (RMSD) of 200 nm×200 nm images taken on the cell top surfaces in the C200 with visible-light (A), the C200 with UV-light (B) and the UV100 with UV-light (C) groups was calculated using image analyzing software to determine the roughness of the cell surfaces. Surface areas with holes and deformations were excluded so that the RMSD data obtained were not affected by the huge changes in topography caused by the holes and deformation sites. The untreated control groups were the bacteria prepared in the same conditions but without illumination. * *P*<0.05, ** *P*<0.01, *** *P*<0.001, compared to the untreated control groups. C200 visible-light: photocatalyzed on C200 by visible light; C200 UV-light: photocatalyzed on C200 by UV light; UV100 UV-light: photocatalyzed on UV100 by UV light.

This study uses AFM to further investigate the bacterial surface changes of roughness. Photocatalyzed bacteria were firstly imaged by AFM, and then the root mean square deviation (RMSD) of 200 nm×200 nm images of the cell top surfaces was calculated using image analyzing software to determine the surface roughness (Fig. 4). In the roughness measurements, surface areas with holes and deformations were carefully excluded so that the RMSD data obtained were not affected by the relatively large changes in topography caused by the hole-like deformations. Since bacteria in different growth phases react differently to chemical antibiotics [18]–[21], we also tested the photocatalytic effects of C200 on all three phases of the bacteria (lag, log, and stationary phases). The roughness of the bacteria surfaces significantly increased within 5 minutes of photocatalysis in all three phases of the bacteria (Fig. 4A, visible light 90 mW/cm^2^; * *P*<0.05, *** *P*<0.001, compared to 0 min untreated groups). Among the three phases, the log phase was most sensitive and significantly affected (Fig. 4A, log phase-1 min groups). Samples catalyzed under UV-light illumination were used as comparisons to determine whether this is a unique feature induced by visible-light stimulated photocatalysis (Fig. 4B, 4C; 2 mW/cm^2^, an UV light density significantly induced antibacterial photocatalysis but without significantly reduced viable bacteria; supplemental Fig. S1, * *P*<0.05, ** *P*<0.01, compared to untreated-dark groups). Because C200 also absorbs energy from UV-light [17], it is not surprising that C200 exhibited the same antibacterial property under UV-light illumination (Fig. 4B, 2 mW/cm^2^). Intriguingly, when compared with the visible-light experiments (Fig. 4A), a similar pattern of roughness change appeared in experimental groups under UV-light illumination (Fig. 4B and 4C, ** *P*<0.01, *** *P*<0.001, vs. untreated groups). Among the three phases, the log phase was most sensitive and seriously affected (Fig. 4B, 4C, log phase-1 min groups). This suggests that a change in surface-roughness is a common response triggered by photocatalysis. Since the roughness analysis was performed on surfaces without obvious deformations, these results suggest that the photocatalysis-mediated change of surface properties coincided with the formation of hole-like deformations.

### Time dependent deformation of *E. coli* cells measured by the AFM

**Figure 5:**
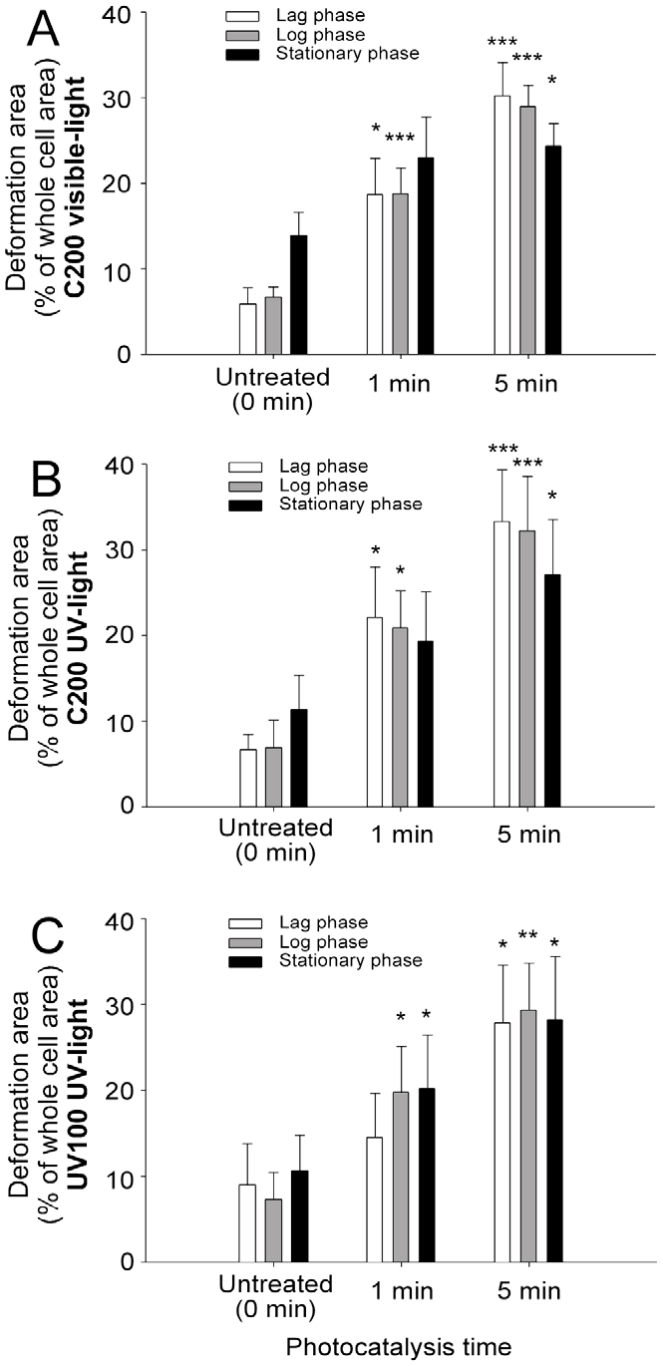
AFM measurements of percentages of deformation area on *E. coli* cells. All three phases of bacteria showed significant increases in the deformation areas in C200 visible-light (A), C200 UV-light (B) and UV100 UV-light (C) groups, under the photocatalysis for 1 and 5 minutes. The untreated control groups were the bacteria prepared in the same conditions but without illumination. * *P*<0.05, ** *P*<0.01, *** *P*<0.001, compared to the respective untreated control groups.

The average deformation area (% of total cell surface area) on the surfaces of bacteria after various treatments was measured (Fig. 5A, C200 visible-light; 5B, C200 UV-light; 5C, UV100 UV-light). The C200 visible-light groups were examined first. The data revealed that the size of the damaged area is positively associated with an increased photocatalysis period (Fig. 5A, visible-light 90 mW/cm^2^; control vs. 1 min and 5 min groups). All three phases of the bacteria showed a significant increase in deformation area under photocatalysis (Fig. 5A, 5 min groups). In agreement with surface-roughness analyses (Fig. 4), the control groups that catalyzed by C200 and UV100 under UV-light exhibited a similar deformation pattern (2 mW/cm^2^; Figure 5B, 5C), as compared with the C200 visible-light groups (Fig. 5A). All three bacteria phases were equally influenced by photocatalysis (Fig. 5A–C, 1 and 5 min vs. untreated). This suggests that UV and visible light-mediated photocatalysis behave similarly in the deformation of bacteria.

### Average deformation position on the bacteria after C200 treatment

**Figure 6:**
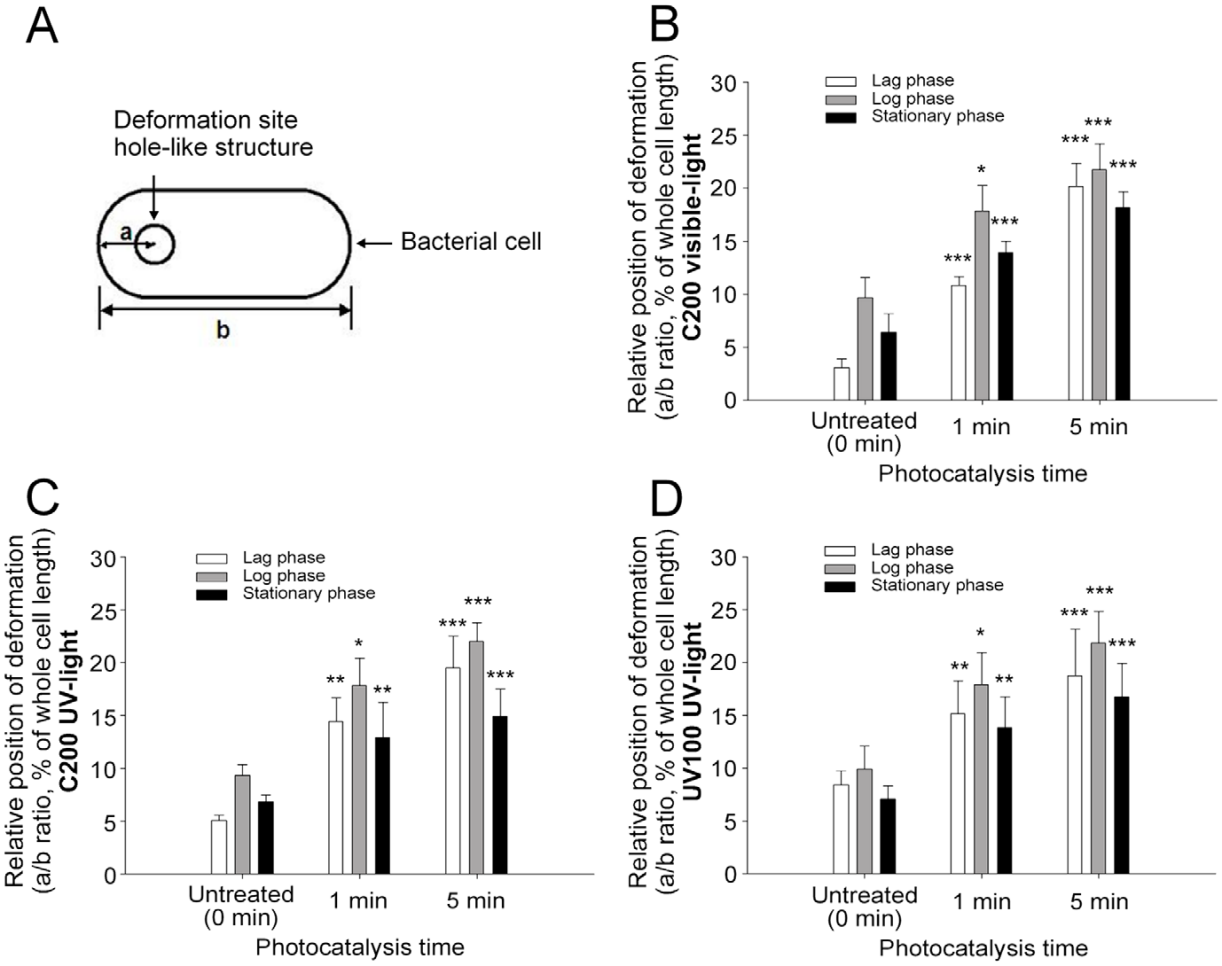
Average deformation centre position on the bacteria after C200-photocatalysis. (A), the deformation center position was identifying by calculating the ratio of the distances between the deformation center and the nearest apical terminus of the bacteria (value a) and the whole bacteria length (value b). (B–D) The average center/bacteria length percentages of the bacteria in C200 visible light (B), C200 UV-light (C) and UV100 UV-light (D) groups, were measured and calculated to be less than 25% in all three phases of the bacteria within 5 minutes of the photocatalysis, indicating that the damage sites were located mainly at the apical terminus of the bacteria. The untreated control groups were the bacteria prepared in the same conditions but without illumination. * *P*<0.05, ** *P*<0.01, *** *P*<0.001, compared to the untreated control groups.

Based on the AFM images above (Fig. 2), the hole-like deformations caused by the photocatalytic reaction seemed to be centered on the two apical terminuses. To quantify these observations, we developed a calculation method to measure the distances between the deformation/hole centers and the nearest apical terminuses of the bacteria (the value “a” in Fig. 6A). The value of “a” was then compared with the whole bacterial length (the value “b” in Fig. 6A). For example, if the deformation was located at the exact center of the bacterium, the center-apical terminus length would calculated as 50% of bacterium length (a/b ratio = 50%). In contrast, for deformations appearing near apical terminus, the “a/b” ratio would be less than 25%. Theoretically, this method may help us determine identify any location-preference of the damage sites, as the apical-terminus preference would lead to a ratio <25%, central preference would lead to a ratio >25%, <50%, and no preference would lead to a random ratio. Intriguingly, the average measurements of the a/b ratio were less than 25% in all groups of photocatalyzed *E. coli* cells (Fig. 6B–D), indicating that there is indeed an apical-terminus preference on the induction of these hole-like deformations. As evidenced by the increasing a/b ratio over time (Fig. 6B–D, untreated vs. 1 min vs. 5 min groups), the center positions of these deformation sites moved progressively toward the central part of the bacteria, implicating the deformations increased during photocatalysis. A similar change pattern on the cellular morphology appeared in all (C200-visible-light, C200-UV-light and UV100-UV-light) groups of photocatalyzed *E. coli* cells, suggesting that the progressive deformation of *E. coli* is common during photocatalysis.

## Discussion

This study shows that AFM is a powerful imaging tool that is capable of achieving nanometer resolution images of biological samples and minimizing the artificial distortions of biological samples. By avoiding harsh chemical or physical treatments, the sample preparations for the AFM imaging are relatively simple compared with SEM. Thus, the distortions of the biological samples can be minimized. Our previous studies use SEM to investigate the damage to photocatalyzed bacterial cells [2], [14], but this approach does not faithfully reveal the hole-like damages, or the dynamics of their formation. Using AFM to study the mechanisms of antimicrobial agents on bacteria, including *E. coli*, has been demonstrated through a series of experiments. For example, antibiotic β-lactam antibiotics cefodizime treatment leads to morphological changes from a standard rod-like morphology into end-to-end fused long filament-like structures on *E. coli* [22]. Another two β-lactam antibiotics, amoxicillin and penicillin, induced randomly formed hole-like structures on bacterial surfaces, although the hole structures and distributions on bacterial cells are distinct among treatments [23]. Other studies provide AFM images of antimicrobial peptide-induced damages to *E. coli* cells [24]–[26]. The results of these studies indicate that antimicrobial peptides also caused randomly distributed abnormal lesions on the cell surfaces. Since photocatalysis mainly produces ROS, which is unlike from the action mechanism of above antibacterial agents, it is not surprising that it produces a different deformation pattern. However, the unique apical-terminus preferential damage found in this study has not been documented yet.

Previous research suggests that ROS is the major antimicrobial agent produced by classical UV-induced photocatalysis. Because visible-light and UV-mediated photocatalysis induced similar deformation pattern, ROS likely plays equally important roles in both UV and visible-light mediated photocatalysis. However, the mechanism of photocatalysis/ROS induced site-specific damages of *E. coli* cells remains unclear. This might be due to the differences in composition between the poles and other parts of the bacteria. The plasma membrane provides a permeability barrier between bacterial cytoplasm and the environments, and is vital to the survival of the bacteria. Maness et al. indicated that bacterial membrane lipid components are likely to be the cellular target of ROS generated by photocatalytic reactions [1]. Using *E. coli* as a model system, the authors found that TiO_2_-mediated photocatalysis promoted peroxidation of the polyunsaturated phospholipid components in bacterial membranes and further led to cell death due to a loss of respiratory activity [1]. If lipid components are the primary targets of the ROS generated by the photocatalytic reactions, the non-homogeneous distributions of lipid compositions in membranes might cause uneven damages of cell membranes, leading to the observation in this study. Indeed, previous reports show that the bacterial plasma membrane lipid distributions are not homogeneous [27]–[31]. The fluorescent dye technique reveals that some lipid did specifically locate at the poles of bacterial cell [29], [31]. According to models proposed by Alley et al. [32] and Mukhopadhyay et al. [33], lipids with relatively unsaturated tails, which create intrinsic curvature of the molecules, tend to concentrated at the poles or apical terminuses of a rod-like bacterial cell to maintain the lowest free energy on the high curvature surfaces. This high concentration of unsaturated lipids forms a lipid disorder phase with high thermo-movement, and eventually creates weak points on the membrane surfaces. This might be the cause for the some small number of deformations found at the poles of the rod shape bacteria even before the photocatalyst treatments. When treated with photocatalyst, the double bonds in the tails of the unsaturated lipids at the bacterial poles caused the bacterial poles to be the primary attacking targets for the ROS generated by the photocatalyst. Once attacked, the double bonds themselves become free radicals and cause a series of chain reactions that make the deformation even more serious [32]–[33]. This might explain the much greater scale of the bacteria pole deformations after photocatalyst treatment.

**Figure 7:**
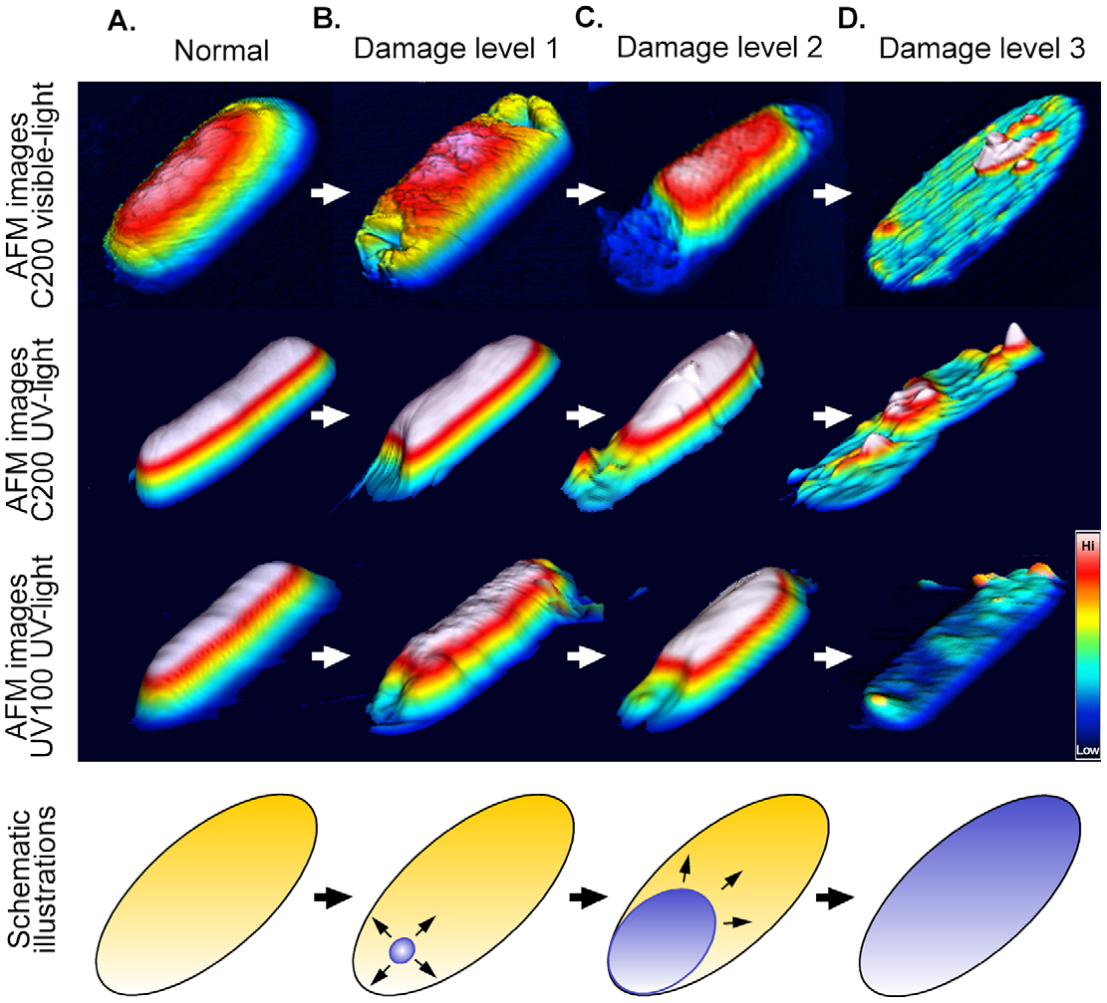
Hypothetical model of photocatalysis-mediated damage progressions in bacteria. Representative AFM images of photocatalyzed bacteria and the schematic illustrations (yellow: undamaged area; blue: damaged area) of various bacterial-damaged states. The pseudo-color in AFM images represents the relative height information of the bacteria; the white color parts represent the higher (Hi) parts, while the black color represents the lower parts (Low). Compared to the normal rod-shaped *E. coli* cells (A), the hole-like structures are preferentially induced near the apical-terminus of the bacterial cells during the initial stage (B) (damage level 1). As the photocatalysis persists, the hole-like structures expand (arrows in schematic illustrations of B, and C) into a larger area and then gradually take flattened shape depicted in (C) and (D) (damage level 2 and 3, respectively). Similar progressive changes of the bacteria were obtained in all three different treatments (C200-visible light, C200-UV light and UV100 UV-light groups).

In addition to lipid components, previous research on the polarity of bacterial cells indicates that polarized expressions of bacteria proteins regulate the pathogenic bacteria-mammalian cell interactions, cell-division, and sporulation. [34]–[35]. Bacterial components, such as the internal scaffold proteins FtsZ and MreB, which are important for maintaining the bacterial cell shape and cell wall synthesis, are preferentially distributed in ring like structures at the middle segment of *E. coli* cells near the cell surface [36]–[38]. Thus it is understandable that such unevenly distributed bacterial components might cause preferential damages on bacterial surfaces. Therefore, this study proposes a hypothetical model of bacterial deformation through photocatalysis (Fig. 7). During the initial phases, the photocatalysis preferentially induces hole-like structures at the terminus poles of bacterial cells, as the membrane components around these positions are easier photocatalysis targets (Fig. 7A–B, normal to damage level 1). Longer and greater damage leads to a leakage of cellular protein components, and the hole-like structures expand into larger areas (Fig. 5, 6; Fig. 7C–D, damage level 2–3). Note that a small amount (<10%) of the untreated cells contains hole-like structures at the poles (Fig. 6B–D, untreated groups). This suggests that these regions are more vulnerable than the other parts of the cells. The cell walls provide a rigid framework and protection for bacterial cells [36], [39]. The data in this study suggests that either the cell wall itself or another unidentified supporting structure underneath the cell wall may rapidly decompose within minutes after photocatalysis. As the damage sites revealed the structural weak points of bacterial cells, future research should investigate the detailed mechanisms of structural maintenance and photocatalysis-mediated deformation in bacteria.

Log phase bacteria, which are actively dividing and are in their optimized growth conditions, exhibited the highest biological and metabolic activities. The majority of antibiotics, including β-lactams and quinolones, require ongoing cell activity and cell division to achieve rapid killing [18]–[21]. Consistently, this study reveals revealed that the bacteria in their log phase were significantly affected by photocatalysis treatments. Results also show that the photocatalysis is highly effective on the bacteria in lag and stationary phases, which are traditionally antibiotic-insensitive. This suggests that antibacterial photocatalysis might have complementary applications with antibiotics.

In summary, this study shows that visible-light driven photocatalysis induced apical-terminus preferential damages and progressive leakages of bacterial protein. This cellular damage is associated with bacterial death. Since the deformation mode of the bacteria is highly associated with the action mechanism of antibacterial agents, these results provide new perspectives on the development of new bactericidal photocatalysts.

## Materials and Methods

### Visible light and ultraviolet light responsive TiO_2_ photocatalysts

The photocatalyst used in these experiments consisted of carbon-containing TiO_2_ nanoparticles prepared using a modified sol-gel method with ethanol as the carbon source. The nanoparticles were subjected to calcination at 200°C (thus named as C200). The details of this process C200 are reported elsewhere [16]–[17], and the photocatalytic studies on C200 have been reported previously [15]–[17]. A commercially available TiO_2_ nanopowder (UV100, Sachtleben, Germany) that can exert the photocatalytic property only when illuminated by UV light was used for comparison [14].

### Photocatalytic reaction and bactericidal effect on *E. coli* cells

*E. coli* (laboratory strain DH5α) was maintained and cultured in Luria-Bertani (LB) broth (Alpha Biosciences Inc., Maryland, USA) or LB agar (Bio Basic Inc., Canada) at 37°C using standard laboratory *E. coli* culture methods [40]–[41]. Bacterial concentrations were determined by the standard plating method and by optical density readings at 600 nm (OD_600_). The factor for converting the OD_600_ values of the bacterial culture to concentration (CFU/ml) was calculated to be 6×10^8^ CFU/ml per OD_600_ by plotting OD_600_ of serial dilutions of fresh bacterial culture against the cell counts of the serial dilution of bacterial culture measured by the standard plating method. To determine the bactericidal effects of the TiO_2_ nanoparticles, 200 µl of overnight culture of bacteria was transferred into 5 ml of culture medium and incubated at 37°C until an OD_600_ of 0.3 to 0.6 (log phase) was reached. The bacterial concentrations were then diluted to 5×10^5^ CFU/ml with culture medium. Fifty microliters of the bacterial culture (2.5×10^4^ CFU) was mixed with 150 µl of normal saline containing the photocatalyst nanoparticles (final concentration 100 µg/mL) and placed on a 24-well cell culture dish (TPP, Switzerland). The cell culture dish was then placed under an incandescent lamp (Classictone incandescent lamp, 60W, Philips, Taiwan) to induce a photocatalytic reaction. Unless specified, visible-light illuminations (bacteria killing) were carried out in a 4°C cold room to control the reaction temperature around 15°C; the *E. coli* population tested herein was not significantly reduced under these conditions. A light meter (model LX-102, Lutron Electronic Enterprises, Taiwan) was used to measure the illumination density. Reaction illuminations were carried out at a distance of 5 cm from the lamp, corresponding to an illumination density of 3×10^4^ lux (lumen/m^2^)(or 90 mW/cm^2^). Ultraviolet (UV) light illumination experiments were carried out at room temperature (25°C) with a light density 2 mW/cm^2^ using an UV lamp (GL15, UV-C; Sankyo Denki Co., Kanagawa, Japan). After illumination, the bacterial solutions were recovered from the 24-well cell culture dishes, and an aliquot of fresh culture medium was used to collect the residual bacteria on the wells of the culture dish. These two bacterial solutions were then combined The bacterial concentration was determined by the standard plating method immediately after bacterial collection, and percentages of surviving bacteria were calculated as described previously [6], [14]. Commercially available TiO_2_, labeled as UV100 (Sachtleben, Germany), and polystyrene latex beads (Sigma-Aldrich, Saint Louis, Mo, USA) were used as controls.

### Bacteria samples for AFM experiments

For AFM studies, one *E. coli* colony was inoculated into 5 ml LB broth. After incubation overnight at 37°C, 0.5 ml of the cultured bacteria was poured into 500 ml LB broth and incubated at 37°C. Based on the bacterial growth curve obtained by optical density measurements at 600 nm (OD_600_), bacteria samples were collected at 2 hours (OD_600_ of approximately 0.05), 6 hours (OD_600_ of approximately 0.7), and 12 hours (OD_600_ of approximately 1) to represent the bacteria in the lag phase, log phase, and stationary phase, respectively. The bacterial cell suspensions were mixed with photocatalysts and illuminated as stated in the previous section. The samples for AFM imaging were collected before illumination and after 1 or 5 min illumination. Data was collected from three independent experiments. In each experiment, 26–36 randomly selected of *E. coli* cells were taken for analyses (sample size: n = 26–36)

### Atomic Force Microscopy

The experiments in this study used a commercial NanoWizard AFM (JPK instrument, Germany) equipped with a liquid 100 µm scanner. The 200 µm long gold coated cantilevers with oxide-sharpened Si_3_N_4_ tips (OMCL-TR400PB-1) used in the experiments were purchased from Olympus, Japan. The spring constant of these cantilevers was 0.02 nN/nm. The mica wafer was cleaned by sonication for 20 min in 1 M HCl, rinsed thoroughly with deionized water, dipped in methanol, and then rinsed again with deionized water to remove debris. The AFM samples were prepared by dropping a diluted bacteria sample onto freshly prepared mica wafer slides. The resulting electrostatic interactions attached the bacteria to the slides. After an adsorption time of 20–40 min, the samples were gently washed with Milli Q water to remove any cells that were not attached to mica. The samples were then air-dried and imaged using contact mode with an applied force of 0.5 nN, as measured by the AFM controller and controlling software. The scan rate was 2–4 lines/sec. The root mean square deviation (RMSD) data was analyzed using image analyzing software provided by NanoWizard AFM (JPK).

### Analysis of protein leakage of bacterial cells

Protein leakage from the bacteria during the photocatalyst treatments was analyzed using sodium dodecyl sulfate polyacrylamide gel electrophoresis (SDS-PAGE) on the proteins outside the bacterial cells. The late log phase bacteria were washed twice and re-suspended in phosphate buffered saline (PBS) before treatment. The bacteria were then mixed with the photocatalyst and illuminated as described in previous sections. The photocatalyzed bacteria and the photocatalyst particles were removed by centrifugation (8000 g, 4°C, 10 mins) at 1 minute and 5 minutes post treatment. The supernatants containing the proteins leaked from the bacteria were then analyzed. The bacterial pellets from respective groups were re-suspended and subjected to sonication (VC 130PB, Sonics & Material, Newtown, CT, USA) to break the bacteria. The bacterial lysate of respective groups was collected by centrifugation (8000 g, 4°C, 10 mins) to measure the residual proteins inside the cells and create an internal control. The collected protein samples were subjected to 15% SDS-PAGE and silver staining (Sigma-Aldrich, USA) to visualize the relative amount of bacterial proteins. Based on a standard curve analysis, the sensitivity of silver staining used in this study reached 2 ng per band. The marker (land M) used in the experiments was the PageRulerTM prestained protein ladder SM0671 (Fermentas, Canada).

### Data Analysis Software

AFM data were collected and analyzed using JPK Imaging Processing Software, version 3.1.6 (JPK instrument, Germany). The statistical differences between groups were calculated using the Student t test and presented as mean ± standard deviation (SD). A *P* value of less than 0.05 (*P*<0.05) was considered statistically significant. Statistical tests were carried out and output to graphs using Microsoft Excel (Microsoft Taiwan, Taipei, Taiwan) and SigmaPlot (Systat Software, Point Richmond, CA, USA) software. Quantitative SDS-PAGE measurements were performed using the public software Image J version 1.41 (National Institute of Health, USA).

## Supporting Information

Figure S1 **Antibacterial properties of UV light responsive photocatalyst UV100 and visible light responsive photocatalyst C200 under UV light illumination.** To evaluate the antibacterial performance of UV100 and C200 photocatalysts (100 µg/mL) under UV-illumination (2 mW/cm^2^, 5 min), the survival rates of *E. coli* cells (total 1×10^4^ CFU in 200 µL solution) were determined. Both UV100 and C200 groups showed significant antibacterial property compared to photocatalyst untreated groups with (UV-light) or without (dark) UV light illumination (* *P*<0.05, ** *P*<0.01, compared to untreated-dark groups). The “dark” groups were the bacteria prepared in the same conditions without illumination. CFU: colony forming unites. The number of surviving bacteria (CFU) in untreated-dark groups was normalized to 100%.
(TIF)
