# Supplementary figures and images for: Visible Light Responsive Photocatalyst Induces Progressive and Apical-Terminus Preferential Damages on Escherichia coli Surfaces
Source: PLoS One. 2011 May 12;6(5):e19982. doi: 10.1371/journal.pone.0019982 (PMC3093399; doi:10.1371/journal.pone.0019982)

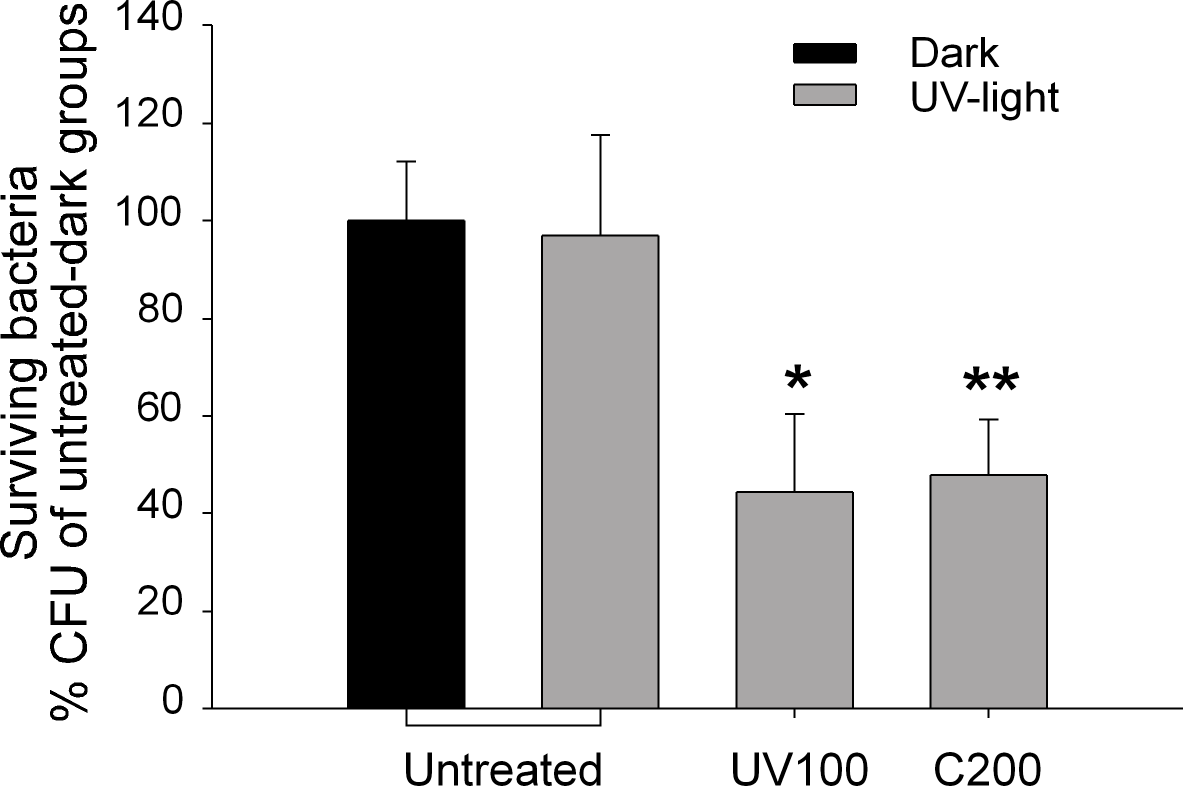

Supplement: Figure S1 — Antibacterial properties of UV light responsive photocatalyst UV100 and visible light responsive photocatalyst C200 under UV light illumination. To evaluate the antibacterial performance of UV100 and C200 photocatalysts (100 µg/mL) under UV-illumination (2 mW/cm2, 5 min), the survival rates of E. coli cells (total 1×10^4^ CFU in 200 µL solution) were determined. Both UV100 and C200 groups showed significant antibacterial property compared to photocatalyst untreated groups with (UV-light) or without (dark) UV light illumination (* P<0.05, ** P<0.01, compared to untreated-dark groups). The “dark” groups were the bacteria prepared in the same conditions without illumination. CFU: colony forming unites. The number of surviving bacteria (CFU) in untreated-dark groups was normalized to 100%. (TIF) [file pone.0019982.s001.tif]
